# Closure of a bronchopleural fistula complicating cryoprobe biopsy of the lung

**DOI:** 10.1002/rcr2.319

**Published:** 2018-04-06

**Authors:** Rahul H. Mehta, Jeffrey Hoag, Amit Borah, Emil Abramian

**Affiliations:** ^1^ Department of Pulmonary and Critical Care Drexel University College of Medicine Philadelphia PA USA; ^2^ Department of Pulmonary and Critical Care Cancer Treatment Centers of America Philadelphia PA USA

**Keywords:** Alveolo‐pleural fistula, bronchopleural fistula, cryoprobe biopsy, Spiration valve

## Abstract

Cryoprobe biopsies are routinely performed by the interventional pulmonologist. Diagnostic yields are larger, with complication rates that are equal to or lower than that of traditional forceps biopsies. We will specifically evaluate one instance where a cryoprobe biopsy led to an alveolo‐pleural fistula that did not resolve with simple tube thoracostomy. An endobronchial valve was placed and successfully resolved the pneumothorax and persistent air leak.

## Introduction

Cryoprobe biopsies are routinely used by interventional pulmonologists to obtain large pieces of bronchial tissue without “crush artefact,” which is often seen on forceps biopsies. Since first being described in 2009 1 in Germany, this technique has gained popularity as diagnostic yields are reportedly greater and complication rates are lower than that of traditional forceps biopsies 2.

Cited complications of cryoprobe biopsy are bleeding and pneumothorax. The latter has been successfully treated with simple tube thoracostomy 1, 2. Despite increased usage of cryoprobes, it appears that cryoprobe biopsies complicated by persistent pneumothorax have gone unpublished. Logically, the larger size of the tissue obtained may suggest increased complication rates compared to traditional forceps biopsies due to the larger calibre of removed tissue. However, few, if any, clinical studies exist evaluating complication rates of cryoprobe biopsies, and the incidence of complications remains largely unreported.

Initially, endobronchial valves were utilized in patients with severe emphysema in an attempt to decrease dead space and areas of ventilation perfusion (V/Q) mismatch in the lung 3. The use of these devices has expanded to include non‐healing communications between airways and the pleural space. As of 2008, Spiration® valves have been given an exemption by the Food and Drug Administration (FDA) and are used on a humanitarian basis. Although still used for severe emphysema, they can now be used for postoperative persistent air leaks, specifically when there are low or no inter‐lobar collateral ventilation (“complete fissures”). Spiration® valves can provide a focal area of atelectasis to an area affected by an abnormal bronchial–pleural communication.

Here, we describe the case of a woman who developed a pneumothorax that complicated the cryoprobe biopsy of a ground‐glass parenchymal infiltrate, which was successfully treated with placement of a Spiration® valve.

## Case Report

**Figure 1:**
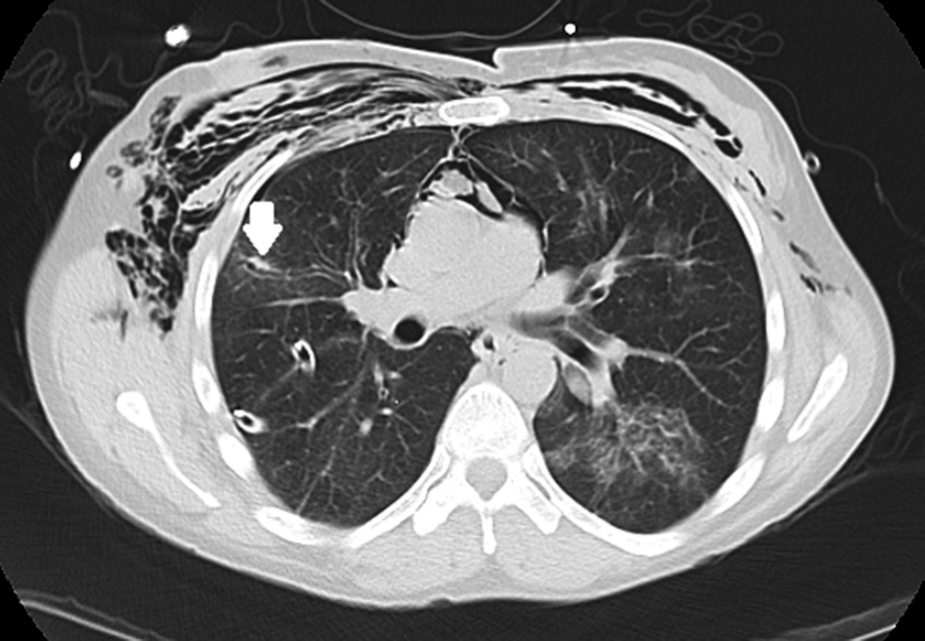
White arrow: sub‐segmental airway distal to right upper lobe, anterior segment, lateral sub‐segment markedly dilated and inflamed.

**Figure 2:**
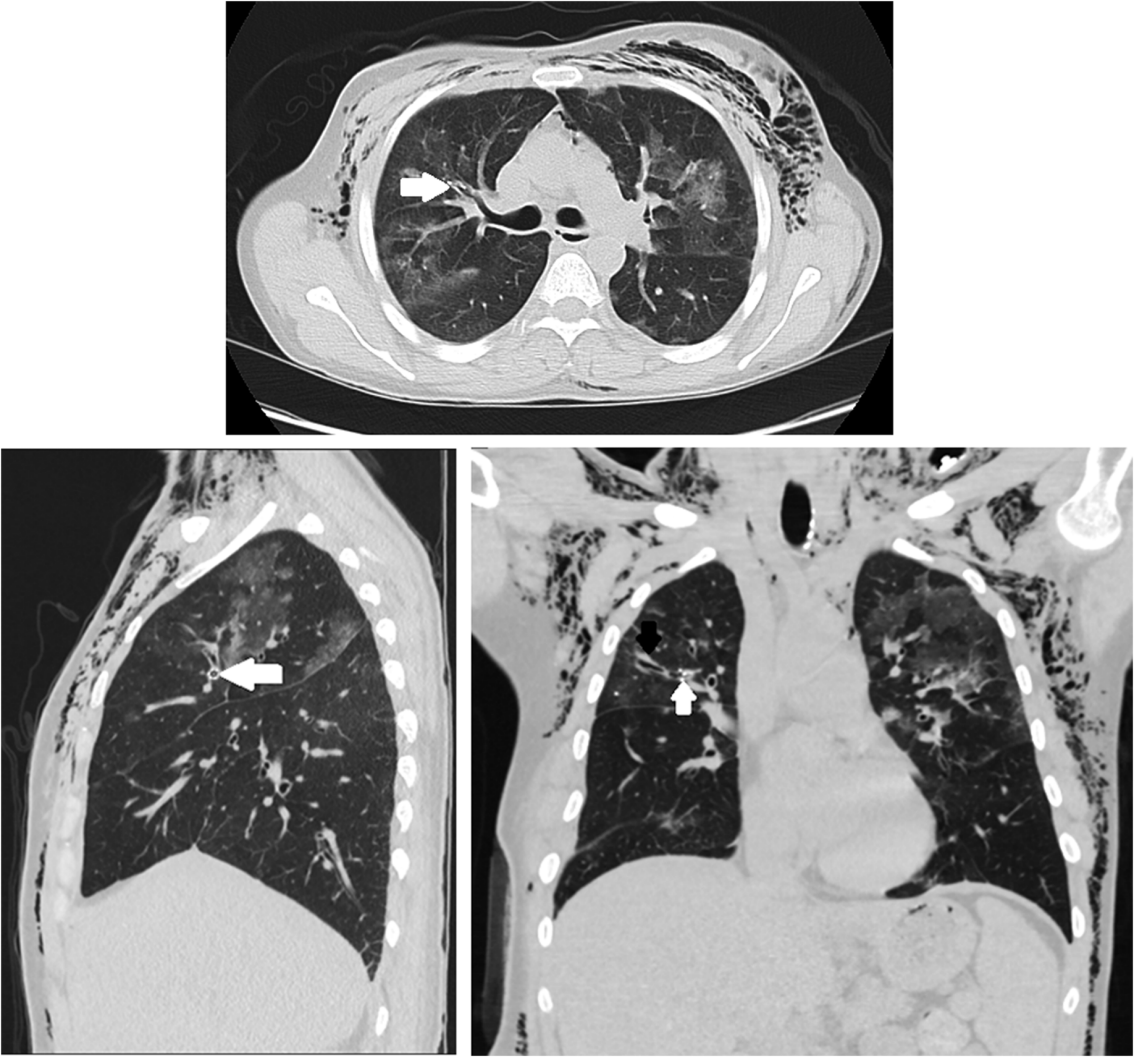
White arrow: Spiration© valve located in lateral sub‐segment of right upper lobe, anterior segment as seen on coronal, sagittal and frontal sections; black arrow: inflamed airway distal to valve.

A 45‐year‐old female smoker with metastatic invasive ductal breast carcinoma presented for evaluation of migratory scattered ground‐glass infiltrates associated with sporadic dry coughing. Two courses of oral antibiotics did not alter symptoms or findings on imaging. Diagnostic bronchoscopy was performed to determine the aetiology. Based on the ground‐glass appearance, a single cryoprobe biopsy of the anterior segment of the right upper lobe was performed using the flexible, 2.4 mm Cryoprobe® produced by Erbe Med (Georgia, USA). Post‐procedural chest radiograph (CXR) was notable for the absence of an immediate post‐procedure pneumothorax. The following day, the patient returned with severe dyspnoea and chest pain that developed after a coughing spell the night following the procedure. CXR revealed a new large right‐sided pneumothorax. Placement of a small bore chest tube using the UreSil® Thora‐Vent™ (Illinois, USA) pneumothorax kit was conducted. Due to a persistent air leak, a large bore chest tube was inserted without improvement of the air leak. Chest computed tomography showed a visible defect (Fig. 1, white arrow) connecting the anterior segment of the right upper lobe (RUL) with the pleural space in the location of the cryoprobe biopsy. Due to the size of the defect and failure of the air leak to close spontaneously after six days, a single 5 mm Spiration© valve was placed into a medial branch of the anterior segment of the right upper lobe, cutting the air leak off from the damaged bronchus (Fig. 2, white arrows and black arrow, respectively). The larger bore chest tube was successfully removed the following day. Removal of Spiration© valve was conducted three months after insertion, without complication or further pneumothorax. Subsequent pathological analysis showed mild fibrosis without evidence of malignancy, consistent with a clinical diagnosis of cryptogenic organizing pneumonia.

## Discussion

Due to an excellent rate of success diagnostically, cryoprobe biopsies have become a remarkable tool for the interventional pulmonologist. According to recent studies, it appears that, when compared with forceps biopsies, cryoprobe biopsies can lead to an up to three or more times greater yield in tissue 1, 2, with complication rates that are similar to that of forceps biopsies 2.

Common complications do include minor bleeding and pneumothorax, with rates being 4.5% and 0–7%, respectively 2. In our case, not only did the patient experience a large pneumothorax, but he also had a persistent air leak, eventually requiring bronchoscopic intervention, with the assistance of a Spiration® endobronchial valve.

Interestingly, the patient did not initially present with a pneumothorax. She remained asymptomatic for hours after the initial cryoprobe biopsy, and a post‐procedure biopsy showed no evidence of pneumothorax. It was not until heavy coughing hours after the procedure did she demonstrate symptoms. We hypothesized two potential causes of this occurrence. The first hypothesis was postulated based on the patients initial bleeding. It is plausible that the patient had a blood clot in an airway proximal to the fistula, causing temporary loss of ventilation to the area of communication. A bout of coughing may have mobilized this clot, allowing air to now flow freely through the fistula into the pleural space. The second hypothesis is that by obtaining a large cryoprobe biopsy of a distal airway, the airway in turn became thin walled and, therefore, weak. The bout of coughing then caused an increasing amount of pressure, and the end of the cough could have caused further damage to the already weakened airway, causing a fistula. In either case, the patient required direct bronchoscopic intervention to close the defect.

Spiration® valves, which were designed with the intent of reducing airflow to isolated lung tissue in patients with severe emphysema, can now be used with similar intent for patients experiencing postoperative, persistent air leaks. Despite being considered off label, Spiration® valves have been allowed by the FDA as a humanitarian use device precisely for this purpose.

### Disclosure Statement

Appropriate written informed consent was obtained for publication of this case report and accompanying images.
